# Prediction of Progression to Severe Stroke in Initially Diagnosed Anterior Circulation Ischemic Cerebral Infarction

**DOI:** 10.3389/fneur.2021.652757

**Published:** 2021-06-18

**Authors:** Lai Wei, Yidi Cao, Kangwei Zhang, Yun Xu, Xiang Zhou, Jinxi Meng, Aijun Shen, Jiong Ni, Jing Yao, Lei Shi, Qi Zhang, Peijun Wang

**Affiliations:** ^1^Department of Radiology, Tongji Hospital, Tongji University, Shanghai, China; ^2^Shanghai Key Laboratory of Artificial Intelligence for Medical Image and Knowledge Graph, Shanghai, China; ^3^Institute of Healthcare Research, Shanghai, China; ^4^Shanghai Institute for Advanced Communication and Data Science/School of Communication and Information Engineering, Shanghai University, Shanghai, China

**Keywords:** ischemic infarction, stroke volume, artificial intelligence, machine learning, severe stroke prediction

## Abstract

**Purpose:** Accurate prediction of the progression to severe stroke in initially diagnosed nonsevere patients with acute–subacute anterior circulation nonlacuna ischemic infarction (ASACNLII) is important in making clinical decision. This study aimed to apply a machine learning method to predict if the initially diagnosed nonsevere patients with ASACNLII would progress to severe stroke by using diffusion-weighted images and clinical information on admission.

**Methods:** This retrospective study enrolled 344 patients with ASACNLII from June 2017 to August 2020 on admission, and 108 cases progressed to severe stroke during hospitalization within 3–21 days. The entire data were randomized into a training set (*n* = 271) and an independent test set (*n* = 73). A U-Net neural network was employed for automatic segmentation and volume measurement of the ischemic lesions. Predictive models were developed and used for evaluating the progression to severe stroke using different feature sets (the volume data, the clinical data, and the combination) and machine learning methods (random forest, support vector machine, and logistic regression).

**Results:** The U-Net showed high correlation with manual segmentation in terms of Dice coefficient of 0.806 and *R*^2^ value of the volume measurements of 0.960 in the test set. The random forest classifier of the volume + clinical combination achieved the best area under the receiver operating characteristic curve of 0.8358 (95% CI 0.7321–0.9269), and the accuracy, sensitivity, and specificity were 0.7780 (0.7397–0.7945), 0.7695 (0.6102–0.9074), and 0.8686 (0.6923–1.0), respectively. The Shapley additive explanation diagram showed the volume variable as the most important predictor.

**Conclusion:** The U-Net was fully automatic and showed a high correlation with manual segmentation. An integrated approach combining clinical variables and stroke lesion volumes that were derived from the advanced machine learning algorithms had high accuracy in predicting the progression to severe stroke in ASACNLII patients.

## Introduction

Cerebral ischemic infarction leads to approximately 80% of stroke. The mortality rate in patients with cerebral ischemic infarction, which is one of the major causes of long-term disability globally, is increasing year by year (1, 2). Magnetic resonance imaging (MRI) is one of the most effective methods for assessing patients with ischemic stroke, and diffusion-weighted imaging (DWI), in particular, has the advantages of diagnosing acute ischemic lesion in the early stage (3, 4). It is necessary to analyze multidimensional information including imaging examination, clinical history, and laboratory tests to make an objective and comprehensive assessment of a patient's condition and provide accurate diagnostic evaluations and treatment plans to reduce disabilities and deaths. It is clinically common that part of the acute cerebral infarction progressed to severe stroke during hospitalization. Therefore, it is meaningful to predict the severity progress of patients with acute ischemic stroke, as it might be quite useful in treatment decision-making and management of prognostic expectations (5).

At present, the critical assessment of patients with acute cerebral infarction often depends on the experiences of physicians, in reference of clinical information (general information, medical history, neurological scores, laboratory examinations), and imaging examinations, but such assessment can be subjective. Artificial intelligence (AI) algorithms can effectively process multidimensional medical data (6, 7), and machine learning, which is one of the most popular techniques in the AI area, has been increasingly adopted in the diagnosis and prognosis of stroke (8–11), such as the automatic segmentation of cerebral infarction lesions, the quantitative analysis of perfusion, and the prediction of stroke prognosis on computed tomography and MRI images. The calculation and prediction results of AI are more reproducible and objective.

Cerebral ischemic stroke is a complicated condition that involves different brain regions and vessels, while anterior circulation ischemic infarction is more common in clinical practice and lacunar infarcts are rare in severe disease condition. Lacunar infarction is small and eventually forms a softened cyst cavity structure, which is often difficult to distinguish from Virchow–Robin spaces. Larger than 15 mm is a giant cavity and even up to 25 mm. The predictive endpoint in our study was the progression to severe stroke, and therefore, we used the following exclusion standard: maximum diameter of infarct ≤ 25 mm.

Therefore, in this study, the initially diagnosed non-severe patients with acute–subacute anterior circulation nonlacuna ischemic infarction (ASACNLII) were included, and machine learning algorithms were employed to predict if non-severe ASACNLII patients would progress to severe stroke during hospitalization.

## Methods

### Study Population

**Figure 1:**
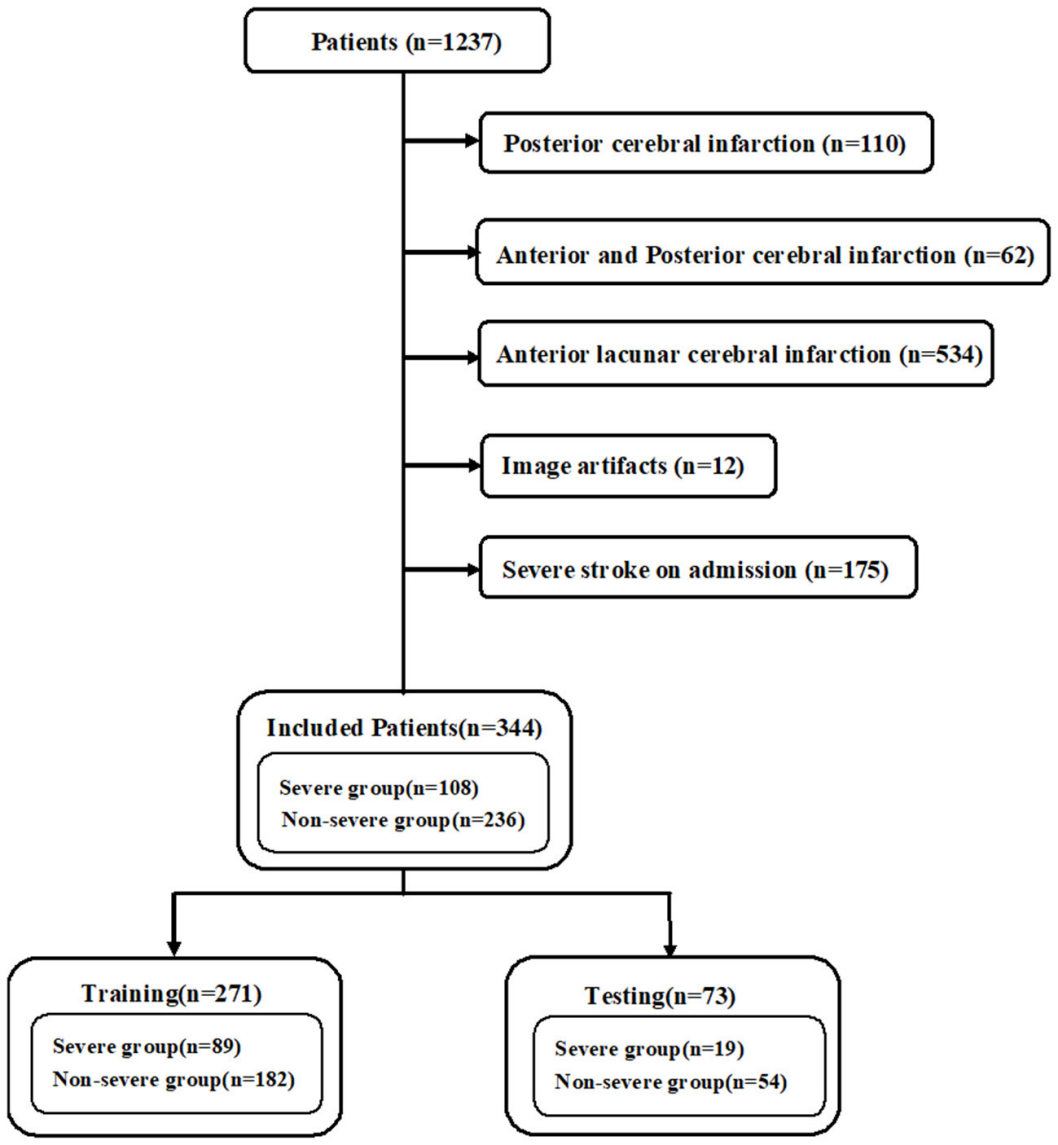
Flow chart illustrating patients selection.

The initially diagnosed patients with ASACNLII who were admitted to the Tongji Hospital, Shanghai, between June 1, 2017, and August 31, 2020 were retrospectively reviewed. The inclusion criteria were as follows: (1) patients who had brain MRI (including MRI-DWI sequence) within 7 days after the onset of symptoms, (2) patients who underwent DWI imaging for depicting lesions with a maximum diameter of >2.5 cm, and (3) initially diagnosed non-severe patients who were admitted to the hospital for treatment. The criteria for non-severe stroke were as follows: National Institutes of Health Stroke Scale (NIHSS) <17; Glasgow Coma Scale (GCS) >8; no hemodynamic instability, no systemic organ dysfunction, no epilepsy, and no mechanical ventilation; and patients with good quality images without any severe artifacts. A total of 1,237 patients with acute–subacute cerebral infarction were included, and 893 patients were excluded due to posterior cerebral infarction (*n* = 110), anterior and posterior cerebral infarction (*n* = 62), anterior lacunar cerebral infarction (*n* = 534), image artifacts (*n* = 12), and severe stroke on admission (*n* = 175). Finally, 344 cases met the enrollment criteria. According to electronic medical records, 108 cases progressed to severe stroke during hospitalization within 3–21 days (Figure 1). The criteria for severe stroke were as follows: NIHSS ≥17, GCS ≤ 8, hemodynamic instability, systemic organ dysfunction, epilepsy, and mechanical ventilation. This study was approved by the institutional review board, and informed consent was exempted due to the retrospective nature of the study. The procedures were performed in accordance with all relevant guidelines and regulations. The subjects were randomly assigned to a training set (*n* = 271) and a test set (*n* = 73). The training set was used for training the AI model and the test set for independent evaluation. The training set was also used for 5-fold cross-validation.

### Data Collection

The MRI-DWI images were obtained using three different MRI scanners. The acquisition parameters were as follows: (1) Philips Ingenia 3.0 T: TR = 2,584 ms, TE = 96.7 ms, slice thickness = 6 mm, slice spacing = 7 mm, field of view = 23 cm × 23 cm, matrix = 256 × 256, excitation times = 2, echo gap = 0.75 ms, *b* value = 1,000 s/mm^2^; (2) Siemens Verio 3.0 T: TR = 4,600 ms, TE = 89 ms, slice thickness = 5 mm, scanning without spacing, field of view = 24 cm × 24 cm, matrix = 256 × 256, echo gap = 0.75 ms, *b* value = 1,000 s/mm^2^; and (3) uMR 1.5 T: TR = 5,400 ms, TE = 94 ms, slice thickness = 5 mm, layer spacing = 6 mm, field of view = 23 cm × 23 cm, echo gap = 0.75 ms, *b* value = 1,000 s/mm^2^.

The following clinical data were collected: (1) general information: sex and age; (2) medical history: history of smoking, alcohol, diabetes, myocardial infarction, coronary atherosclerosis, atrial fibrillation, hypertension, and stroke; (3) neurological score scale: NIHSS and GCS on admission; and (4) laboratory tests on admission: prothrombin time (PT), fibrinogen, D-dimer, serum troponin I, blood glucose, blood lipids, and plasma brain natriuretic peptide (BNP).

### Lesion Segmentation and Volume Measurement on MRI-DWI

#### Image Segmentation and Labeling

The segmentation task was completed by three junior radiologists (Lai Wei 144 cases; Kangwei Zhang 100 cases; Yun Xu 100 cases), and two senior radiologists refined the segmentation results (Aijun Shen 124 cases; Jiong Ni 120 cases). The radiologists segmented and refined the ischemic lesions on MRI-DWI images with ITK-SNAP software (Version 3.8.0, http://www.itksnap.org). Manual labeling was used as the supervisor or teacher of the AI-based automatic segmentation model.

#### Image Preprocessing and Augmentation

The DWI image with a *b* value of 1,000 s/mm^2^ was normalized to the grayscale range, which was defined by the window width and window level. The images at each cross-section were resampled to a size of 256 × 256 pixels. As the amount of data used to train the model was relatively limited, this study used online data augmentation, which included two parts: (1) morphological transformation: −10°~10° rotation around the *z*-axis, 0.95~1.05 scaling, −0.1~0.1 times translation along the *x* and *y* directions, respectively, and left and right mirror transform with 50% probability; and (2) grayscale transformation: linear contrast transformation of 0.8~1.2 times, brightness change of 0.8~1.2 times, and Gaussian blur with a sigma of 0.5.

#### U-Net Model and Training

A convolutional neural network model called the U-Net was designed to accomplish the automatic segmentation of cerebral infarction on MRI-DWI images. The U-Net is a popular AI segmentation model in the medical field. Each DWI image was scaled to a size of 256 × 256, and the U-Net has yielded the lesion masks of the same size. The 3D segmentation mask of a lesion was obtained by stacking the masks of all slices.

**Figure 2:**
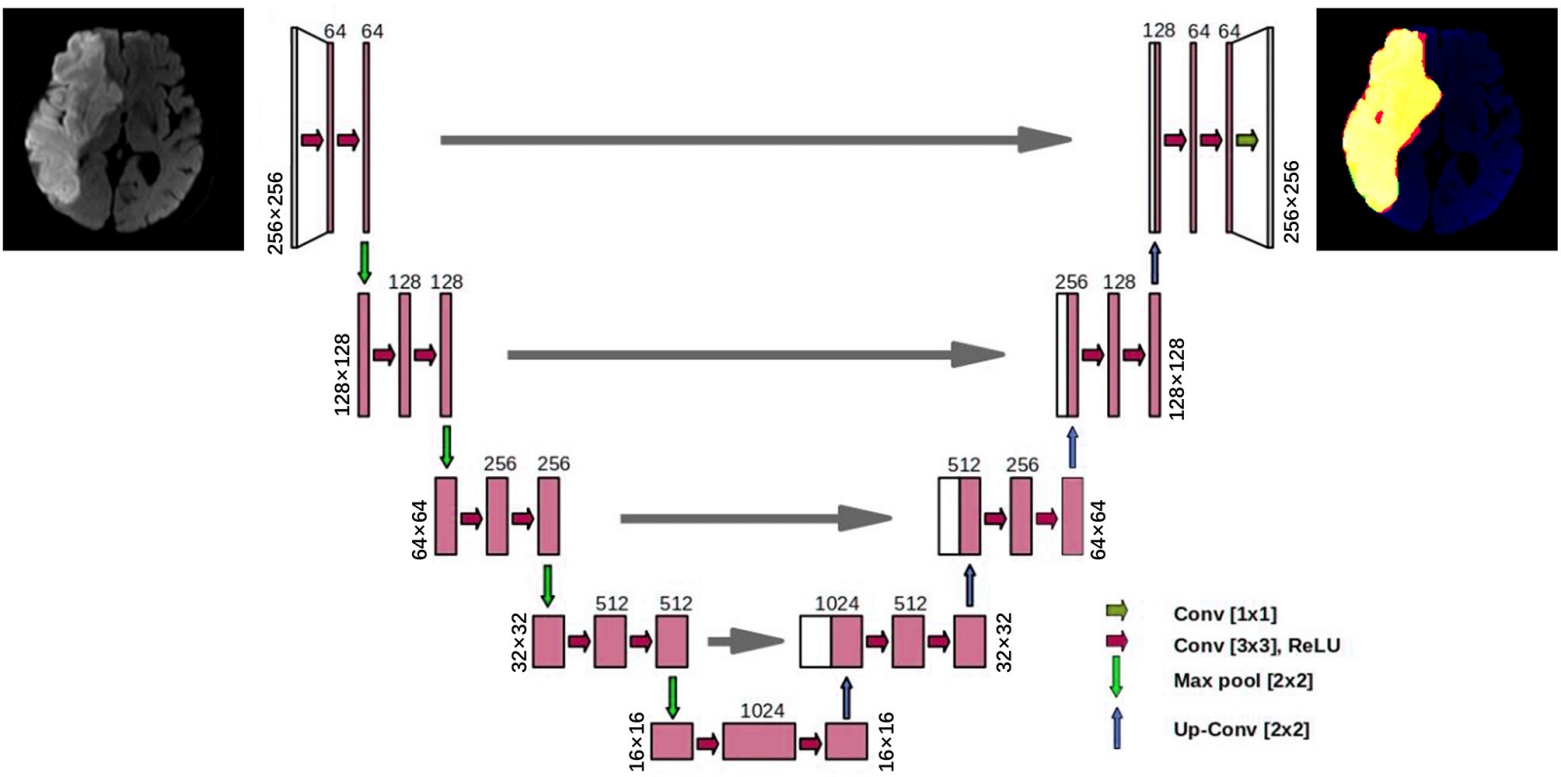
The architecture of the proposed U-net model.

The U-Net model had a total of four down-sampling convolutional layers and four up-sampling transposed convolutional layers (Figure 2). The feature maps of the same resolution were connected by concatenation to integrate the shallow features and deep features. Cosine annealing scheduler was set as the learning rate strategy, with a period of 50 epochs. The minimal learning rate was set to 0.00001 and the initial learning rate was set as 0.01. Stochastic gradient descent was used as the optimizer in the model, and it had a weight attenuation coefficient of 1e−8 to prevent overfitting. The batch size of the model training was 4, and a total of 200 epochs were adopted for training.

### Predictive Model

#### Predictive Task

The predictive task included those initially diagnosed non-severe patients with ASACNLII who progressed to severe stroke during hospitalization. In this retrospective study, the information with regard to patients who were initially diagnosed and their treatment records were obtained from the electronic medical record system. The enrolled patients were divided into a group of patients who progressed to severe stroke (*n* = 236) and a group of patients who did not progress to severe stroke (*n* = 108) according to their medical history. The patients who progressed to severe stroke were all transferred to the neurology intensive care unit (N-ICU) for further treatment according to medical history record.

#### Development and Validation of the Predictive Model

Based on the patients' clinical data and/or AI-derived volume data, three machine learning models were constructed for binary classification (yes/no for progressing to severe stroke) by using three classifiers, namely, random forest (RF), support vector machine (SVM), and logistic regression (LR).

The input data of the prediction model were one of the three feature sets: (1) AI-derived volume data (1 variable), (2) clinical data (19 variables), and (3) volume + clinical combination (20 variables).

The training phase consisted of two stages. In the first stage, the whole training set was separated into training and validation subsets, in a 5-fold cross-validation manner. The hyperparameters were optimized according to the cross-validation experiments. In the second stage, we applied the optimal hyperparameters on the whole training set to train the models, and the computed metrics on the test set were reported. The procedure was similar to traditional training, validation, and test set separation. The test set did not help on hyperparameter optimization.

### Statistical Analysis

Unpaired Student's *t*-test and chi-square test were used for evaluating significant differences in the variables (such as age, NIHSS score, etc.) between the training set and the test set. The Dice coefficient was used to evaluate the performance of AI-based automatic segmentation. The squared Spearman correlation coefficient (*R*^2^) was used to assess the consistency between the lesion volume obtained by AI and the gold standard volume as measured by the radiologists. The receiver operating characteristic curve (ROC) was drawn, and the sensitivity (SEN), specificity (SPE), accuracy (ACC), Youden's index (YI), and the area under the curve (AUC) were calculated for evaluating the model performance.

In addition to the independent evaluation on the test set, the 5-fold cross-validation was also performed on the training set (12). The Shapley additive explanation (SHAP) diagram for the test set was drawn for model explanation. Bootstrapping was used to compute the confidence intervals in the test set. DeLong's method was used to compare the ROCs of different predictive models. A *p*-value lower than 0.05 was considered to be statistically significant. The segmentation task, the training and validation of the predictive model, as well as the statistical analysis, were all programmed by Python (version 3.6).

## Results

### Basic Characteristics

As shown in Table 1, the basic variables of most of the patients showed no statistical differences (*p* > 0.05) between the training set and the test set, such as general conditions (gender and age), medical history (hypertension, diabetes, etc.), neurological score scales (NIHSS and GCS), and laboratory tests (BNP, etc.).

**Table 1:** Basic patient information.

	**Training set (*n* = 271)**	**Test set (*n* = 73)**	***p*-values**
**Basic characteristics**			
Age, median (IQR)	71 (63, 82.5)	75 (66, 84)	0.093
Male (percentile: %)	179 (66.1%)	25 (53.4%)	0.064
**Neurological score scale, median (IQR)**			
NIHSS on admission	6 (3, 12)	8 (4.5, 12)	0.062
GCS on admission	15 (13, 15)	15 (14, 15)	0.338
**History (percentile: %)**			
Alcohol	91 (34.2%)	15 (20.55%)	0.036
Smoking	132 (49.6%)	21 (28.8%)	0.002
Myocardial infarction	9 (3.4%)	1 (0.0%)	0.610
Coronary atherosclerosis	54 (20.3%)	15 (20.5%)	0.906
Atrial fibrillation	43 (16.2%)	17 (23.3%)	0.215
Hypertension	188 (70.7%)	52 (71.2%)	0.958
Stroke	73 (27.4%)	22 (30.1%)	0.759
Diabetes	95 (35.7%)	23 (31.5%)	0.596
**Laboratory test, median (IQR)**			
Prothrombin time	11.0 (10.6, 11.6)	11.1 (10.7, 11.6)	0.090
Fibrinogen	2.83 (2.43, 3.56)	2.75 (2.35, 3.76)	0.278
D-dimer	0.66 (0.34, 1.67)	0.75 (0.33, 1.81)	0.494
Serum troponin I	0.01 (0.01, 0.03)	0.01 (0.01, 0.03)	0.222
Blood sugar	6.46 (5.41, 9.01)	7.65 (5.54, 10.11)	0.051
Blood lipids	1.25 (0.94, 1.75)	1.27 (0.92, 1.62)	0.346
Brain natriuretic peptide	83.7 (38.1, 231.8)	131.6 (49.9, 286.9)	0.070
**Severe progress**			
Progress to severe stroke (percentile: %)	89 (33.0%)	19 (26.0%)	0.322

*IQR, interquartile range; NIHSS, national institute of health stroke scale; GCS, glasgow coma scale*.

### Performance of U-Net

**Figure 3:**
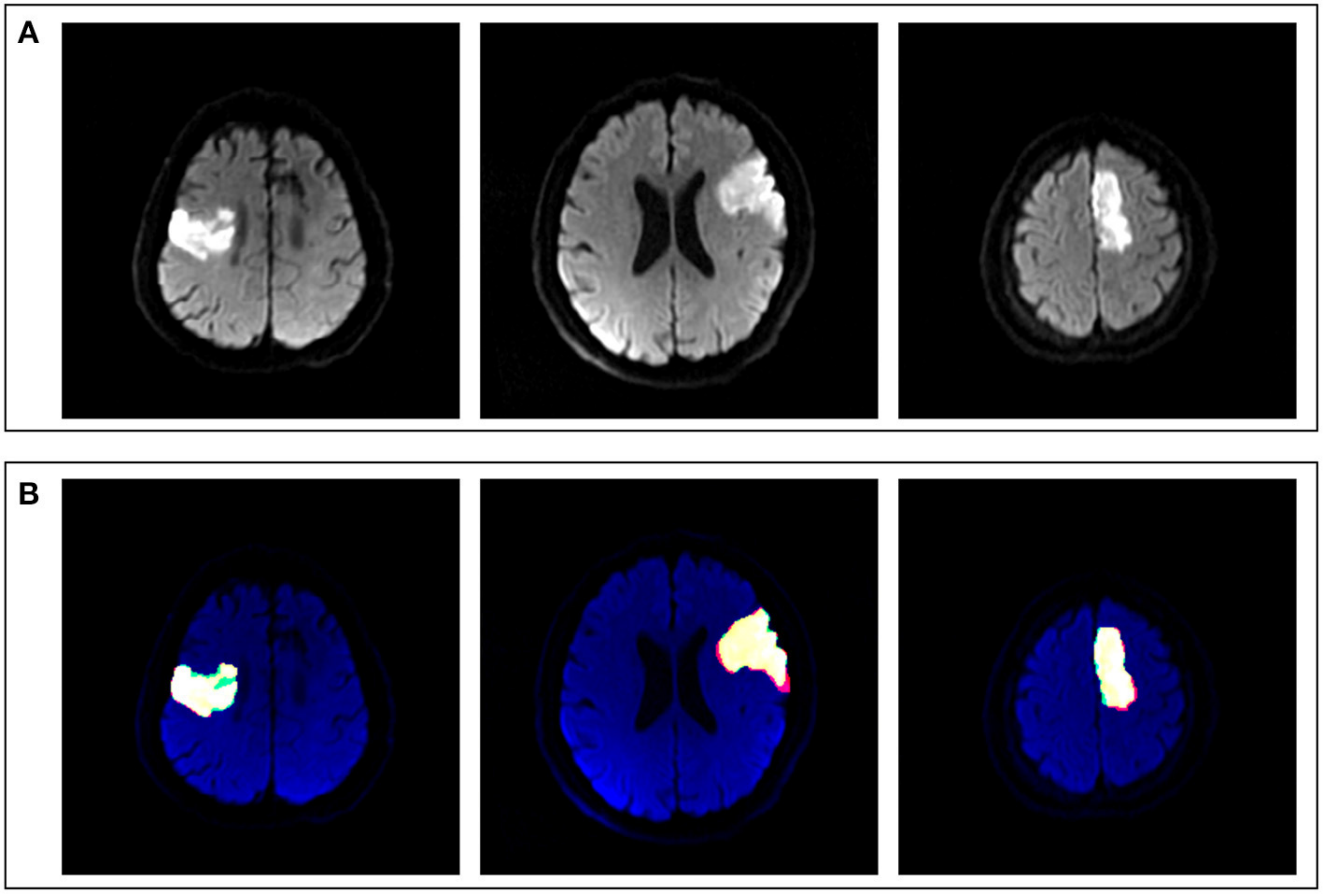
Comparison between artificial intelligence (AI)-based segmentation and manual segmentation in three cases of ASACNLII. **(A)** DWI images. **(B)** Segmentation results, in which red represents manual labeling results, green the AI output results, and yellow the consistent areas.

**Figure 4:**
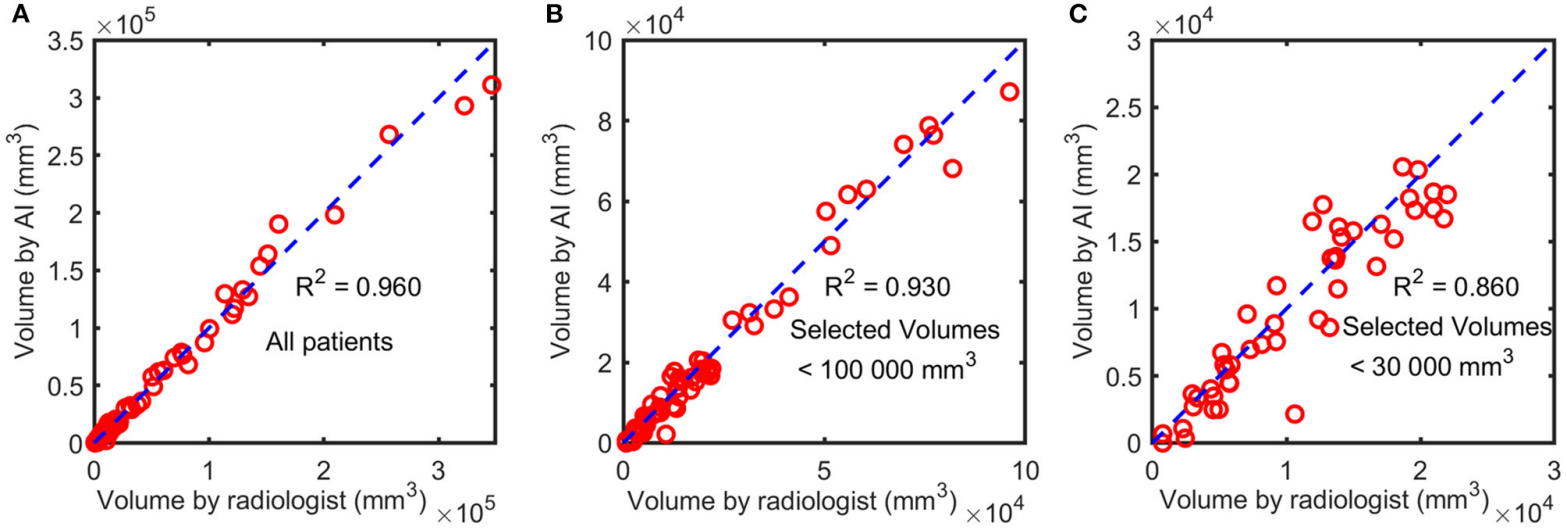
The correlation between AI-derived volumes and the manually segmented volumes. The squared correlation coefficients *R*^2^ were calculated for all patients **(A)**, patients with lesion sizes <100,000 mm^3^ **(B)**, and those with lesion sizes <30,000 mm^3^ **(C)**.

The U-Net achieved highly consistent results of segmentation in the test set when compared with the manual labeling (Figure 3), and the Dice coefficient was 0.806. The correlation coefficient *R*^2^ between AI-derived volumes and the manual segmentation volumes was 0.960 (*p* < 0.0001) (Figure 4A). The grouping by lesion sizes (<100,000 and <30,000 mm^3^) yielded the *R*^2^ values of 0.930 and 0.860, respectively (Figures 4B,C).

### Comparison of Predictive Models

Predictive models were constructed by using the three feature sets (volume data, clinical data, and volume + clinical combination) and the three classifiers (RF, SVM, and LR). The RF classifier-based model using the volume + clinical combination achieved the best predictive classification with an AUC of 0.8358 (95% CI 0.7321–0.9269). Therefore, a comparative analysis was made as follows: (1) fixing the RF classifier and comparing the three feature sets and (2) fixing the combination and comparing the three classifiers.

#### Comparison Between Different Feature Sets

**Figure 5:**
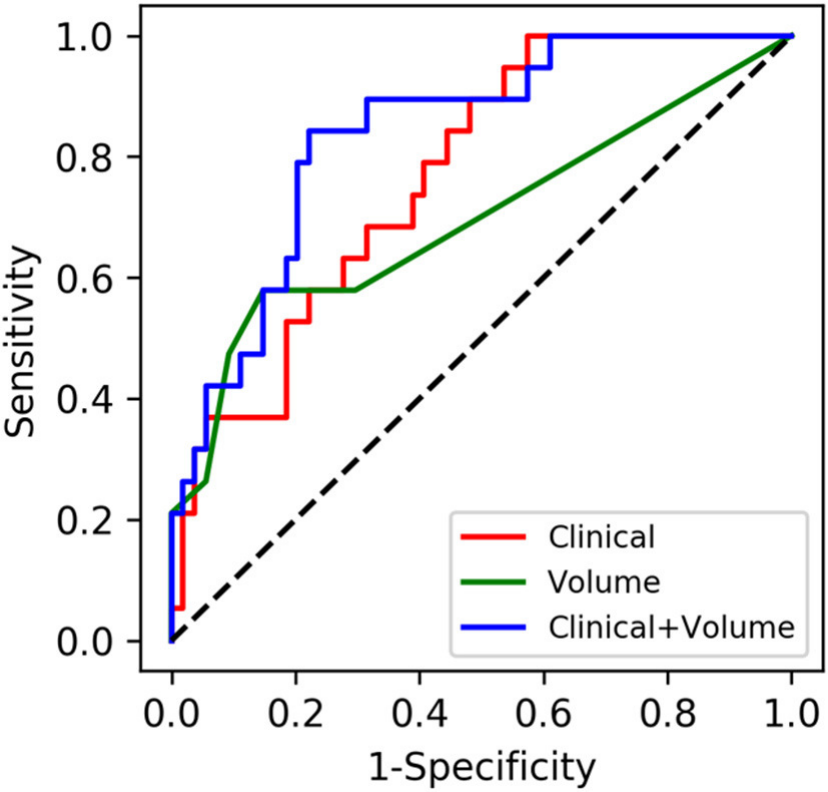
Performances of machine learning models for the prediction of progression to severe stroke: receiver operating characteristic (ROC) curves of three feature sets when using the random forest classifier.

The AUCs of the models using the RF classifier with the clinical data, the volume data, and the combination on the test set were 0.7686 (0.6474–0.8717), 0.6929 (0.5434–0.8262), and 0.8358 (0.7321–0.9269), respectively (Table 2), which were close to the out-of-bag AUCs of 0.7829, 0.6147, and 0.8110 and the 5-fold cross-validation AUCs of 0.8113, 0.7105, and 0.8291 for the clinical data, the volume data, and the combination, respectively. The predictive model of the combination data showed the highest AUC on the test set when compared with the clinical data (*p* = 0.036) and AI-derived volume data (*p* = 0.048) by the DeLong test (Figure 5). The SEN, SPE, and YI of the combination data on the test set have reached 0.7695 (0.6102–0.9074), 0.8686 (0.6923–1.0), and 0.6380 (0.4475–0.8182), respectively.

**Table 2:** Comparison between different feature sets.

	**Clinical data**	**Volume data**	**Clinical + volume combination**
AUC^*^	0.7686 (0.6474–0.8717)	0.6929 (0.5434–0.8262)	0.8358 (0.7321–0.9269)
Sensitivity	0.6022 (0.3607–0.9630)	0.8735 (0.7636–1.0)	0.7695 (0.6102–0.9074)
Specificity	0.8891 (0.5217–1.0)	0.5612 (0.3000–0.7857)	0.8686 (0.6923–1.0)
Youden's index	0.4913 (0.3494–0.6522)	0.4347 (0.1897–0.6628)	0.6380 (0.4475–0.8182)
Accuracy	0.6386 (0.5753–0.7945)	0.7846 (0.7808–0.7945)	0.7780 (0.7397–0.7945)

* *AUC, area under the receiver operating characteristic curve*.

#### Comparison Between Different Classifiers

As shown in Table 3, when fixing the clinical + volume combination, the AUCs of RF, SVM, and LR on the test set were 0.8358 (0.7321–0.9269), 0.8165 (0.6854–0.9344), and 0.8104 (0.6952–0.0.9113), respectively. The ROCs between different classifiers on the test set were compared in pairs, and the results showed no statistical differences (all *p* > 0.05). The hyperparameter optimization of the three machine learning models was searched out in a cross-validation way, and thus, the hyperparameters were finally set as follows: (1) for SVM, C = 0.01, kernel = “rbf,” degree = 3, gamma = “scale,” probability = True, random_state = 1, decision_function_shape = “ovo,” max_iter = −1, verbose = 1, tol = 0.0001, and class_weight = {0:2, 1:1}; (2) for LR, C = 1, random_state = 1, solver = “lbfgs,” multi_class = “multinomial,” max_iter = 5,000, penalty = “l2,” verbose = 1, and class_weight = {0:181, 1:90}; and (3) for RF, criterion = “entropy,” bootstrap = True, random_state = 1, oob_score = True, n_estimators = 100, max_features = 1, max_depth = 6, min_samples_split = 3, min_samples_leaf = 1, and class_weight = {0:181, 1:90}.

**Table 3:** Comparison between different classifiers.

	**Logistic regression**	**Support vector machine**	**Random forest**
AUC^*^	0.8104 (0.6952–0.9113)	0.8165 (0.6854–0.9344)	0.8358 (0.7321–0.9269)
Sensitivity	0.7226 (0.4074–0.9815)	0.8631 (0.6471–0.9608)	0.7695 (0.6102–0.9074)
Specificity	0.8200 (0.5000–1.0)	0.8013 (0.6000–1.0)	0.8686 (0.6923–1.0)
Youden's index	0.5426 (0.3680–0.7363)	0.6644 (0.4657–0.8644)	0.6380 (0.4475–0.8182)
Accuracy	0.7103 (0.5616–0.8219)	0.8334 (0.6849–0.8493)	0.7780 (0.7397–0.7945)

* *AUC, area under the receiver operating characteristic curve*.

#### Model Interpretability

**Figure 6:**
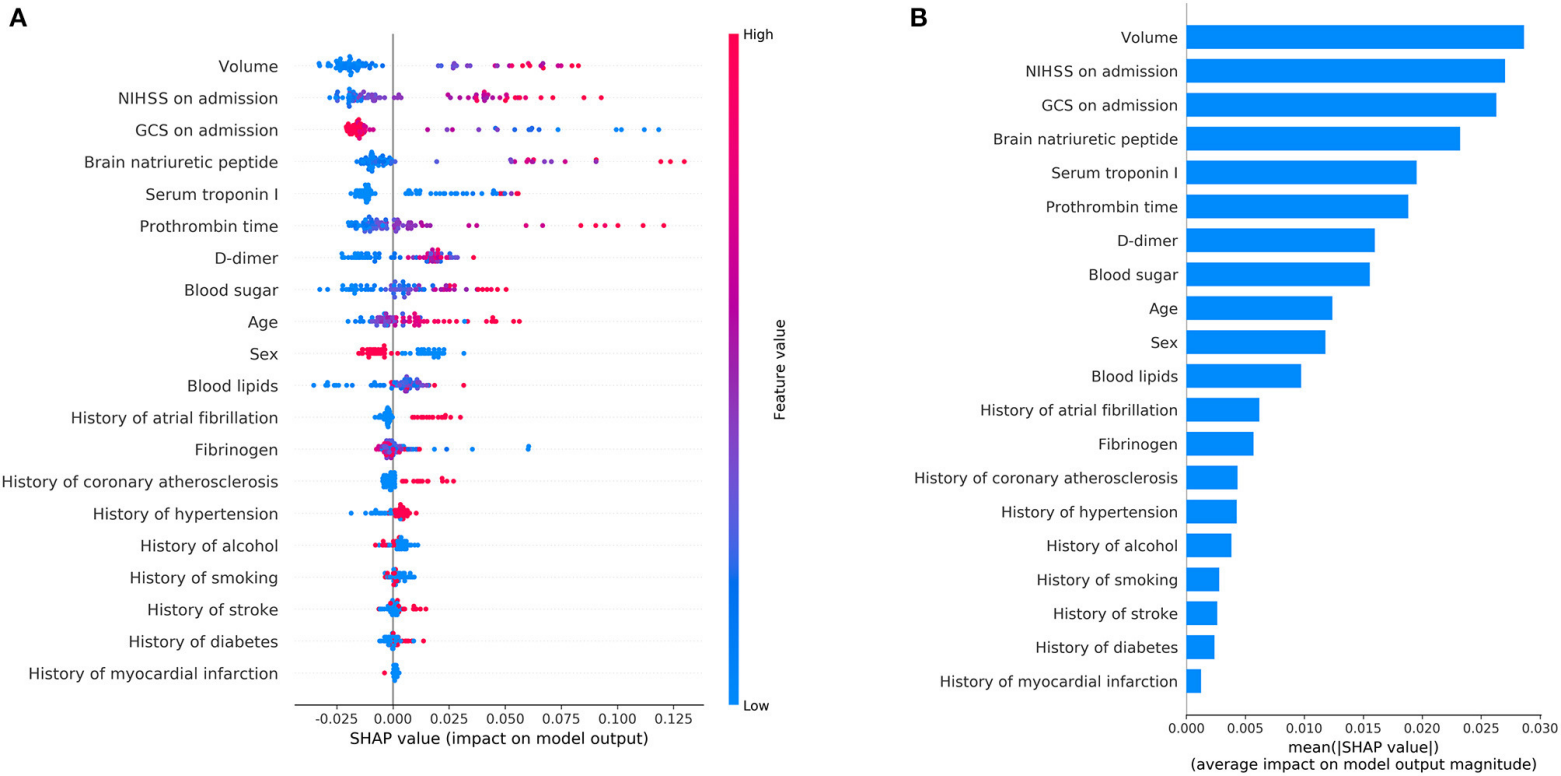
Shapley additive explanation (SHAP) diagram of variable contributions for the optimal predictive model, i.e., the random forest classifier with volume + clinical data. **(A)** The relative contributions of AI-derived volumes and clinical variables for progression prediction. Features on the right of the risk explanation bar pushed the risk higher, and features on the left pushed the risk lower: a patient with a larger volume, higher NIHSS, and lower GCS is at a higher risk. **(B)** The relative contributions of variables for progression prediction quantified with the mean of the absolute SHAP values.

**Figure 7:**
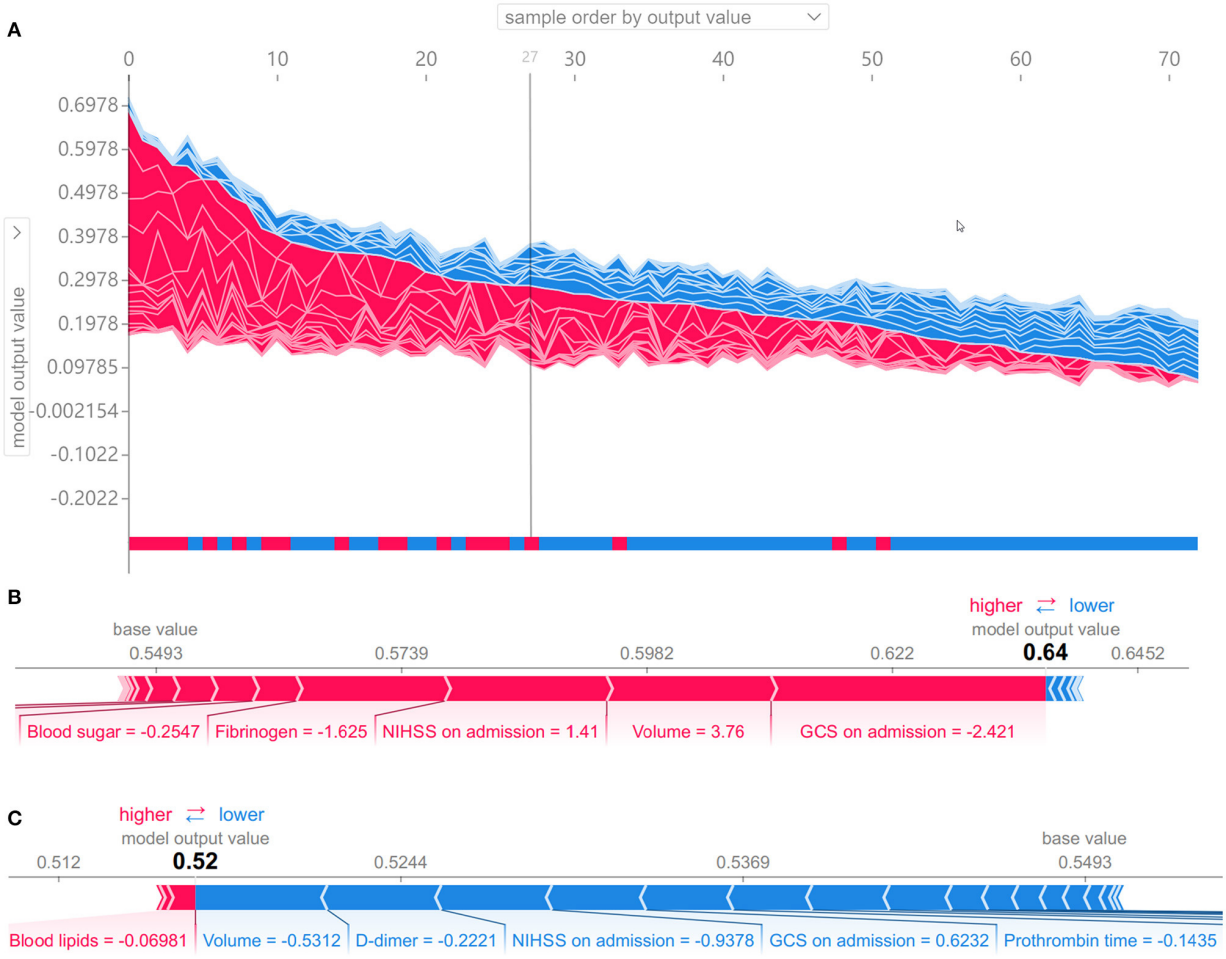
Shapley additive explanation (SHAP) values to show interpretability of the effects of AI-derived volumes and clinical variables as the input risk factors for the prediction of progression to severe stroke. **(A)** SHAP values for all 73 patients in the test set. Samples from left to right are ordered by the sum of the SHAP values from all variables, and the bottom bar shows the true labels of each sample, namely, red for the positive group (progression to severe stroke) and blue for the negative group (non-progression to severe stroke). The 27 samples on the left are predicted as positive samples by the random forest model. **(B, C)** SHAP values of two typical patients from the positive group **(B)** and the negative group **(C)**, illustrated with their most important variables.

As shown in Figure 6, the SHAP diagram of the above optimal predictive model, namely, the RF classifier with the clinical + volume combination, showed that cerebral infarction volume was the most important predictor in severe stroke progression. The NIHSS and GCS on admission also played an important role in this predictive model, and BNP acted as an important biochemical indicator of severe stroke progression for ASACNLII. In addition, other conventional biochemical indicators and age contributed to the predictive model. However, gender and medical history factors showed slight significance in this predictive model. Moreover, SHAP values for all 73 patients in the test set were shown in Figure 7A. The detailed SHAP values of the most important variables for one typical patient in the positive group (progression to severe stroke) and one in the negative group (non-progression to severe stroke) are illustrated in Figures 7B,C. These figures further demonstrated that the AI-derived volume serves as an essential risk factor for prediction of progression to severe stroke.

## Discussion

Our study used a U-Net deep learning model for lesion segmentation. The Dice coefficient was boosted from 0.680 to 0.790 by diverse data augmentation, and cosine annealing learning rate scheduler was used to further improve the Dice coefficient to 0.806, indicating that the data augmentation method was essential for enhancing the segmentation performance. The high quality of automatic segmentation led to high accuracy of subsequent lesion volume measurement with an *R*^2^ value of 0.960. The infarction volumes on DWI were combined with the multidimensional clinical information for more accurate prediction of progression to severe stroke. The results of this study revealed that the lesion volume of ASACNLII was the most important predictor as illustrated in the SHAP diagram and this was consistent with the literature and clinical practice (12, 13). The predictive model of clinical information (AUC = 0.7686) also showed good performance, and the AUC of the predictive model on volume + clinical combination using RF was as high as 0.8358, which was better than the model that used the volume data or clinical data alone (*p* < 0.05). The difference of AUC was very small between using the AI-predicted volume as a predictor (0.8358) and using the radiologist's volume (0.8387). It was not surprising because the AI-predicted volume was very close to the radiologist's volume (squared correlation coefficient *R*^2^ = 0.960).

The three factors that contributed the most were the lesion volume, NIHSS on admission, and GCS on admission, and this was in concordance with the current consensus (14–16).

The AI-based segmentation methods of cerebral infarction areas on DWI images were divided into two main categories: (1) the thresholding methods: Lee et al. have reported that the correlation coefficient between the thresholding method of the infarct core area and the gold standard of manual segmentation was 0.62 ± 0.18 (17); Boldsen et al. have developed a thresholding method on DWI for acute anterior circulation stroke with a median Dice coefficient of 0.3951 (18). (2) Deep learning methods: Nishi et al. have reported that the Dice coefficient of the U-Net model of the core infarct area was 0.58 ± 0.01 (19); Kim et al. have reported that the average Dice coefficients of infarct region segmentation based on U-Net for DWI + ADC and DWI were 0.60 and 0.57, respectively (20); and Wu et al. have reported that a deep learning segmentation model achieved a Dice coefficient of 0.86 (0.79–0.89) (9). These previous studies have indicated that the AI methods have increased popularity in DWI lesion segmentation, and the AI algorithms, especially the deep learning approaches, can accurately and automatically trace the lesion border of ischemic stroke.

Imaging findings can be used as an input feature, as well as a supplement to clinical features, for predicting stroke prognosis or outcome. Vogt et al. have reported that the initial lesion volume of cerebral infarction acted as an independent predictor of prognosis (90d-Rankin score) (13). Heo et al. have built a deep learning model of clinical information including general information, medical history, and laboratory tests to predict the prognosis of patients with acute cerebral infarction (yes/no 90d mRS: 0–2), and the area under the ROC curve has reached to 0.81 ± 0.06 (21). Lee et al. have revealed that the prediction of 6-month swallowing recovery was feasible based on clinical and radiological factors using the Bayesian network model, and their study also emphasized the importance of bilateral subcortical lesions as prognostic factors as these could be utilized to develop prediction models for long-term swallowing recovery (22). However, combining both lesion volumes and clinical data for predicting the progression to severe stroke has not yet been reported.

Our study has built a machine learning model to accurately predict the progression to severe stroke in initially diagnosed nonsevere patients with ASACNLII. It is quite essential for treatment planning, preparing transfer to the N-ICU, and effective communication between doctors and patients. A meta-analysis study has shown that the transfer to N-ICU has significantly reduced the mortality and improved the prognosis of stroke patients, while not all patients required transfer to the N-ICU from the perspective of health economics (14). Accurate prediction of progression to severe stroke can provide clinical evidence and help prepare for transfer to the N-ICU in order to obtain better therapeutic efficacy for patients with high risks. In addition, the AI-based predictive model provides a more objective reference for treatment decision, and it would be particularly helpful for the medical staff who do not specialize in stroke.

The data were randomly partitioned into training and test sets in a ratio of 3:1. There were 19 variables in the basic patient information, and it was very difficult to generate random sets which had no significant differences in any of the 19 variables between the training and test sets. Finally, we chose a partition which had no significant differences in 17 of the variables (*p* > 0.05) and only the history of smoking and the history of alcohol exhibited significant differences (*p* < 0.05). Previous studies showed that smoking and alcohol use were complicated epidemiological risk factors of stroke. Smoking increased the risk proportionally with the number of cigarettes smoked per day (23), and heavy alcohol use and acute alcohol ingestion increased the risk of stroke, especially hemorrhagic stroke (24). In these studies, the behaviors of smoking and alcohol use were quantified or semiquantified, but in our study, we only retrospectively collected the history of smoking or alcohol as binary variables of medical history information without quantification. Hence, we speculated that these two variables of patient history should have a minor effect on our results of prediction of severe stroke progression.

This study has some limitations. Firstly, the data collection had a single-center geographic limitation, which cannot represent the overall distribution of the disease in a wide range of population. The predictive model was trained and fitted based on the data generated by this particular center. Data from other sources should be collected for external validation. Secondly, this study collected multidimensional data, such as basic information, image information, and clinical information, and this was time-consuming, resulting in a small sample size. However, compared with previous studies with regard to the diagnosis and prediction of cerebral infarction using deep learning technology, the sample size is basically the same (25–27). Thirdly, despite high average AUCs, the confidence intervals were relatively large, which showed model instability and needed further fine-tuning on larger datasets to improve its stability. Lastly, when collecting data, lacunar cerebral infarction patients with good prognosis were excluded, which meant that this study did not include all clinically common cases of cerebral infarction.

## Conclusions

The U-Net for infarction lesion segmentation is fully automatic and shows a high correlation with manual segmentation. A machine learning approach using both clinical and volume feature sets has demonstrated a high accuracy for the prediction of the progression to severe stroke in initially diagnosed non-severe patients with ASACNLII, and hence, it has good potential for clinical application and guarantees further clinical validation in larger samples.

## Data Availability Statement

The raw data supporting the conclusions of this article will be made available by the authors, without undue reservation.

## Ethics Statement

The studies involving human participants were reviewed and approved by the Ethics Committee of Tongji Hospital (approval number: K-2020-021). Written informed consent for participation was not required for this study in accordance with the national legislation and the institutional requirements.

## Author Contributions

PW made substantial contributions to the design of the work. LW, KZ, JM, and JY accomplished the acquisition of data. LW, KZ, and YX finished the segmentation labelling task. AS and JN refined the labelling results. LW, YC, and QZ built the segmentation and predictive models. LW accomplished the analysis, interpretation of the work, and drafted the manuscript. QZ, LS, and PW revised it for important intellectual content. All authors have approved the final version to be published.

## Conflict of Interest

The authors declare that the research was conducted in the absence of any commercial or financial relationships that could be construed as a potential conflict of interest.

## Abbreviations

ASACNLII: acute–subacute anterior circulation nonlacuna ischemic infarction
SHAP: shapley additive explanation
RF: random forest
SVM: support vector machine
LR: logistic regression.
